# Frailty and audiovisual senses in older patients with diabetes: a cross-sectional observational study

**DOI:** 10.20407/fmj.2022-029

**Published:** 2023-08-28

**Authors:** Sayuri Sable-Morita, Yuko Harasawa, Kiyomi Yamada, Saiko Sugiura, Hideki Fukuoka, Haruhiko Tokuda

**Affiliations:** 1 Department of Nursing, National Center for Geriatrics and Gerontology, Obu, Aichi, Japan; 2 School of Healty Sciences, Toyohashi Sozo University, Toyohashi, Aichi, Japan; 3 School of Nursing, Seirei Christopher University, Hamamatsu, Shizuoka, Japan; 4 Toyota Josui Mental Clinic, Toyota, Aichi, Japan; 5 Department of Otorhinolaryngology, National Center for Geriatric and Gerontology, Obu, Aichi, Japan; 6 Department of Ophthalmology, Kyoto Prefectural University of Medicine, Kyoto, Kyoto, Japan; 7 Department of Clinical Laboratory/Department of Endocrinology and Metabolism, National Center for Geriatric and Gerontology, Obu, Aichi, Japan

**Keywords:** Diabetes, Older, Frailty, Hearing loss, Visual impairment

## Abstract

**Objective:**

This study aimed to analyze the relationship between frailty in older patients with diabetes and audiovisual senses.

**Methods:**

The survey included (1) basic attributes, (2) diabetes-related items, (3) frailty, evaluated according to the Obu Study of Health Promotion for the Elderly (OSHPE) standard, and (4) audiovisual function. Participants included 157 diabetes patients aged ≥65 years, divided into three groups: robust health (n=50), pre-frail (n=76), and frail (n=31). A simple regression analysis, in which the total OSHPE score was used as the dependent variable and the most relevant audiovisual items were used as independent variables, was performed to analyze the frailty factor. Next, a multiple regression analysis adjusted for age and sex was performed with total OSHPE score as the dependent variable and the items most relevant for audiovisual senses as independent variables.

**Results:**

For the robust health, pre-frail, and frail groups, frequencies of hearing loss were 18.4%, 42.1%, and 35.5%, respectively, and were associated with frailty; visual impairment frequencies were 38%, 63.2%, and 58.1%, respectively. In multiple regression analysis, economic difficulties (B=0.349, β=0.172, p<0.05), absence of dyslipidemia (B=–0.494, β=–0.171, p<0.05), lower MNA score (B=–0.169, β=–0.214, p<0.05), and worsening hearing in the poor hearing ear (B=0.015, β=0.176, p<0.05) were significantly associated with frailty.

**Conclusions:**

Hearing but not vision was associated with frailty in older patients with diabetes.

## Introduction

In recent years, the relationship between frailty and diabetes in older people has received increasing research attention. The concept of frailty is defined by several factors, including muscle weakness, and is also a predictor of poor outcomes, such as impaired life functioning, death, hospitalization, falls, fractures, and cognitive decline caused by age-related physiological decline,^1,2^ indicating a preliminary step toward the state of needing long-term care. Hyperglycemia in older diabetic patients is a risk factor for frailty, and is associated with reduced muscle mass and medication use.^3^ Complications of frailty are reported to shorten life expectancy.^4,5^ Older diabetic patients with frailty have higher rates of hospitalization and mortality than those without frailty, often requiring attention and management, including glycemic control with medications and nutritional guidance.^6^

Sensory organ function is essential for activities of daily living in older individuals. Audiovisual and frailty assessments are considered to be necessary for the prevention of cognitive decline in older individuals,^7^ and the concept of sensory frailty has been proposed. Both vision impairment and hearing loss are associated with frailty.^8^ Regular ophthalmic examinations are recommended for retinopathy, a complication associated with diabetes. Hearing loss is more frequent in older diabetic patients.^9^ A follow-up study of approximately 140,000 women showed a higher risk of hearing loss in those with type 2 diabetes compared with those without diabetes, and those with diabetes ≥8 years in duration were at a higher risk of developing moderate or worse hearing loss.^10^ In Japan, Uchida et al.^11^ examined the relationship between diabetes treatment and hearing loss in 90 middle-aged and older diabetes patients and reported that hearing deteriorated as the duration of diabetes increased.

Risk factors for frailty in older diabetic patients include age, low levels of albumin, high-density lipoprotein cholesterol, systolic blood pressure, glycated hemoglobin (HbA1c), total cholesterol, and weight.^12^ However, reports on frailty in older diabetic patients focusing on audiovisual function are scarce.

This study aimed to analyze the relationship between frailty in older diabetic patients and audiovisual sensory function.

## Methods

### Participants and study design

This cross-sectional observational study included patients with diabetes aged ≥65 years who did not qualify for long-term care insurance and visited the Diabetes Outpatient Department of Center A from October 2015 to May 2016. This study was only able to enroll 157 patients who underwent an audiovisual test during the period mentioned above.

### Research items

Individual characteristics, including age, sex, height, weight, body mass index (BMI), diabetic complications, and HbA1c level, were obtained from electronic medical records. Additionally, inquiries were made on social background factors and whether they experienced economic difficulty in life. Cognitive function was evaluated using the Mini-Mental State Examination (MMSE) as an item for older functional evaluation.^13^ The MMSE has a full score of 30, with ≥24 categorized as normal, <10 as severe decline, and <20 as intermediate decline. For nutritional evaluation, the Mini Nutritional Assessment (MNA) was used. Participants who scored ≥12 points were categorized as well-nourished.^14^ Participants who scored ≤11 were classified as possibly undernourished, a 12-item assessment was performed, and the score was summed with a screening score (maximum 30 points) for evaluation. Usage of insulin, dipeptidyl peptidase-4 (DPP-4), sodium-glucose cotransporter 2 (SGLT2), glucagon-like peptide 1 (GLP-1), sulfonylurea (SU), pioglitazone (Pio), and biguanide (BG) were obtained from electronic medical records.

### Frailty

The Obu Study Health Promotion for the Elderly (OSHPE) criteria,^15^ including five items from Fried et al.,^16^ were used to evaluate frailty. The measurement methods for the five items were modified for feasibility in Japan. The validity and reliability of the OSHPE were verified in 5,104 community-dwelling people aged ≥65 years. The number of items that applied to each participant was counted from the following five items: “weight loss,” “fatigue,” “decreased physical activity,” “decreased grip strength,” and “decreased walking speed.” For “Weight loss,” 1 point was given if patient responded “yes” and 0 points were given if the patient responded “no” to the following question: “Have you lost more than 5% of your weight in the last 2 years?” For “fatigue,” 1 point was given if the response was “yes” and 0 if “no.” For “Physical inactivity” 1 point was scored for “not doing light exercise/exercise, regular exercise/sports” and 0 for “doing light exercise/exercise, regular exercise/sports.” If the response to the question regarding “Decrease in grip strength” was “no,” 1 point was scored for “male: <26 kg, female: <17 kg,” and 0 points for “male: ≥26 kg, female: ≥17 kg.” The grip strength of the dominant hand was measured three times, and the average value was used. The grip strength meter used was Smedley’s Dynamometer No. 30548. “Walking speed decrease” was 1 point for “male: <1.0 m/s, female: <1.0 m/s” and 0 points for “male: ≥1.0 m/s, female: ≥1.0 m/s.” Normal walking speed was used for walking speed measurements. A 1-m runway was made before and after the measurement section, and the time taken to walk the 5-m measurement section was recorded. By summing all five items, “frail” was defined by the presence of three or more applicable items, while individuals with one or two applicable items were categorized as “pre-frail” and those with no applicable items were categorized as “healthy.”

### Measurement of vision

#### Simple visual acuity measurement

A near vision chart was used for the simple visual acuity test, and bilateral visual acuity was measured 30 cm away. If necessary, customary corrections were used, such as eyeglasses and contact lenses. The cutoff value for the left or right eye was set to 0.5 according to the distribution, and participants were categorized into two groups: <0.5 (visual impairment) and ≥0.5 (without impairment).^17^ Visual acuity assessments from the cohort were converted into log MAR units.

Regarding the subjective evaluation of poor vision, we asked participants if they were aware of their vision loss using the question, “Do you find it difficult to see?”, then asked if they used eyeglasses and/or had a history of cataract surgery.

### Measurement of hearing

A pure tone hearing test was performed using an audiometer (model number AA-78 manufactured by Rion Co., Ltd., Tokyo, Japan). The AA-78 had an inspection sound level of –20–105 dB, and bilateral hearing was measured twice at frequencies of 500, 1,000, 2,000, and 4,000 Hz. A soundproof room was used for testing. In the finger rub hearing test, the dry index finger and thumb were rubbed together to make a sound from the back, and the presence or absence of the participant’s reaction was examined. For presentation of the finger rub sound, the inspector stood 30 cm behind the participant and performed a finger rub with a volume of approximately 40 dB; the distance between the fingertip and ear was 5 cm. A sound level meter (Rion Co., Ltd., NL-20) was used in advance to confirm that the finger rubbing sound of the inspector nurse was approximately constant at approximately 40 dB.^9^ This test was performed twice on both sides of the right and left ears. For participants who regularly used hearing aids, detection of finger rubs was measured while they were using their hearing aids.

Participants were asked the question, “Do you think you have difficulty hearing?” Responses were given as either “yes” or “no.” Regarding the evaluation of hearing by others, participants were asked the question, “Have others mentioned that you have poor hearing?” Participants responded with “yes” or “no” regarding their hearing loss (referred to hereafter as an indication of hearing loss). Finally, we asked participants if they were using hearing aids.

The definition of moderate hearing loss or higher according to World Health Organization (WHO) standards was used. Better average hearing of 500, 1,000, 2,000, and 4,000 Hz sounds that exceeded 40 dB was considered hearing loss.

### Analytical methods

On the basis of the OSHPE criteria, participants were divided into three groups according to the level of frailty (healthy, pre-frail, and frail), and comparisons of the survey items among them were analyzed using the chi-square test for nominal variables^18^ or the Kruskal-Wallis test^19^ for continuous variables.

For visual function, we analyzed the history percentage of cataract surgery. The left and right hearing levels were categorized as good or poor, and descriptive statistics were calculated.

The rates of diabetic complications and insulin use were analyzed for each group.

A simple regression analysis, in which the total OSHPE score was used as the dependent variable and the most relevant audiovisual items were used as independent variables, was performed to analyze the frailty factor. Next, a multiple regression analysis adjusted for age and sex was performed with the total OSHPE score as the dependent variable and the items most relevant for audiovisual sensory function as independent variables. Furthermore, a multiple regression analysis was performed after adjusting for age and sex with the total OSHPE score as the dependent variable and all of the items found to have an association in univariate and regression analyses as independent variables. The most dominant items in vision status and hearing status were entered into the regression analysis. SPSS version 22 was used for the statistical analysis, with a significance level of 5%.

## Results

Of the 200 diabetes patients aged ≥65 years who visited Center A, this study included 157 (79 males and 78 females) who underwent audiovisual evaluation (Table 1).

Frail diabetic patients were significantly older, had more economic difficulties, and lower MMSE and MNA scores than non-frail diabetic patients.

Participants’ height and weight decreased from the healthy group, to the pre-frail group, to the frail group. The three major complications were neurosis, retinopathy, and nephropathy, occurring in 75 (47.4%), 46 (29.3%), and 87 (55.4%) patients, respectively. The proportion of neurosis tended to increase, leading to frailty in 31.8%, 48.4%, and 19.7% of participants in the healthy, pre-frail, and frail groups, respectively. There were significant associations between OSHPE score and height, weight, and dyslipidemia.

There was no association between diabetes medications and frailty, and no patients were using SGLT2. No frail patients were using Pio.

Of the 157 patients with near vision (cutoff value of 0.5), 85 (54.1%) had visual impairment. More than 95.5% of participants had a history of cataract surgery on either the left or right side; 49 (31.2%) participants were subjectively aware of vision loss, and 78 (49.7%) used eyeglasses. Visual evaluation revealed a significant association between the three frailty groups and visual impairment. Compared with patients in the healthy group, 63.2% and 58.1% of participants were visually impaired in the pre-frail and frail groups, respectively.

Moderate to high hearing loss according to the WHO classification was observed in 8 (24.2%), 31 (32.3%), and 13 (48.1%) patients in their 60s, 70s, and 80s, respectively.

The three frailty groups significantly differed in the percentage of participants with poor hearing, finger rub hearing test performance loss, WHO hearing loss, and indication of hearing loss. There was a significant difference in the frequency of all hearing measures between robust, pre-frail, and frail groups, in all evaluation methods.

Further analysis was performed, focusing on the hearing ability of the poor hearing ear that showed the highest correlation with frailty for each audiovisual sense item. In univariate analysis, the poor hearing ear value was significantly associated with the OSHPE score (Table 2 Model 1). Regression analysis with OSHPE as the dependent variable with a cutoff of 0.5 and acuity as the input variable showed no significant differences (Table 2, Model 2).

In Model 3, regression analysis was performed with OSHPE score as the dependent variable, age and gender as adjustment variables, and poor hearing ear value, simple vision cutoff value of 0.5, neurosis, economic difficulty in life, dyslipidemia, cerebrovascular disease, MNA score, and MMSE score as independent variables.

Economic difficulties (B=0.349, β=0.172, p<0.05), absence of dyslipidemia (B=–0.494, β=–0.171, p<0.05), MNA (B=–0.169, β=–0.214, p<0.05), and worsening hearing in the poor hearing ear (B=0.015, β=0.176, p<0.05) were significantly associated with frailty (Table 2, Model 3).

## Discussion

The current study investigated the correlation between frailty and audiovisual sensory function in older adults with diabetes and diabetic complications, and identified factors that contribute to frailty in patients with diabetes. The results of this study indicate that poor hearing, dyslipidemia, MNA score, and economic difficulties are associated with frailty in older adults with diabetes.

In the current study, the factors associated with frailty in older diabetic patients were not only basic attributes like age, and sex, or diabetes-related factors like insulin and other drug use, HbA1c and other values, and the presence of diabetes-related complications, but also focused on audiovisual impairment. Additionally, the methods of assessing audiovisual impairment were examined using multiple models. We examined several methods for evaluating audiovisual impairment. When these were examined in the input model, a strong association with frailty was found in the presence or absence of hearing loss in the poor hearing ear.

Reports show a significant association between frailty and hearing loss.^20^ The link between hearing loss and social frailty has been reported among older women in the South Korean population.^21,22^

Hearing loss is common in older people with diabetes,^9^ and is associated with a tendency toward frailty, with decreased input of sound information, cognitive decline, depression, and social isolation.^23^ Additionally, hearing loss reduces social participation and interaction. Decreased outings and other activities because of social frailty are also associated with muscle weakness and physical frailty, and people with hearing loss are currently more likely to become frail. In other words, older people with diabetes are at increased risk of frailty because of hearing loss.

Because a person with normal hearing in one ear but poor hearing in the other ear may have a lower subjective evaluation of their hearing,^24^ although they may be reluctant to go out because of their concerns about their poor hearing, and as a result, may be more likely to become frail.

Previous literature reviews have focused on the impact of good hearing. In one-on-one conversations, if good hearing is present in one ear, there is typically no difficulty in overall hearing. However, in noisy situations, if a person has hearing loss in one ear, significant hearing difficulty is often experienced. With aging, a person’s good hearing ear may also develop age-related hearing loss, increasingly requiring repetition of speech. Cochlear microangiopathy caused by diabetes can cause hearing loss, and the auditory system, which has high energy demands because of complex signal processing, is more likely to be a target of hyperglycemic adverse effects.^3^

Because subjective evaluation is sometimes inaccurate in older individuals, we conducted an objective evaluation. However, because pure tone audiometry is time-consuming, we also performed the finger rub test, in the hope that the results would also show a significant association. Although the final model included the presence or absence of hearing loss in the poor hearing ear on the basis of pure tone audiometry, univariate analysis showed that finger rub test performance was significantly associated, providing a potential surrogate measure.

Regarding visual impairment, a cutoff value of 0.5 is used to assess visual acuity in many countries, and has been reported in various guidelines. An association between dementia and a visual impairment cutoff value of 0.5 has been reported in many studies.^25,26^ Although there is a paucity of literature assessing frailty and objective visual acuity, a 5-year longitudinal study evaluating the association between self-reported frailty in older people reported a 2.5-fold incidence of frailty with self-reported visual impairment.^27^ In the present logistic regression analysis, visual impairment and frailty based on a cutoff of 0.5 were not significantly associated, but it is possible that objective longitudinal evaluation would reveal an association.

The incidence of frailty tends to increase with age. Various complications may occur with a long-term history of diabetes, resulting in a poor prognosis and further promotion of frailty.

In the current study, frailty was associated with economic difficulties and undernutrition (Table 2). Additionally, we found a relationship between economic difficulty and MNA scores, and each was independently associated with frailty.^28^

In older people, nutritional balance tends to be poor, and the frequency of dyslipidemia tends to decrease.^29^ In a study examining the prevalence and income of diabetes in 119,666 adults, the risk of diabetes increased 1.9-fold in males^25^; although this report was not specifically focused on older people, low income is also likely to be associated with undernutrition and diabetes in older populations.

The causes and background of undernutrition in older people include decreased oral intake, decreased activity, and various overlapping background factors, such as comorbidity, medication, decreased oral function, psychological changes, social environment (including living alone and in households with older persons), and economic problems such as poverty.^30^ Further attention should be paid to diabetes patients because dyslipidemia, undernutrition, and financial distress that increase with age are independently associated with frailty.

Because the current study employed a cross-sectional design, the findings should be investigated further using longitudinal evaluation. Additionally, because the sample population consisted solely of older adults with diabetes who consume alcohol, comparisons with the broader community of older adults will be necessary to establish the generalizability of the current findings.

The strengths of this study included a focus on audiovisual senses to elucidate the factors associated with frailty in older patients with diabetes, thorough measurement of physical functions, and an analysis of complications. Thus, the factors mentioned above strengthened our study of physical aging in older patients with diabetes, which is an underlying factor in frailty. Further clarifying the lifestyle and physical and social factors associated with frailty in this population will improve current understanding of its causes.

## Conclusion

Hearing but not vision was associated with frailty in older people with diabetes.

## Disclosure

The authors declare no conflict of interest.

## Figures and Tables

**Table1:** Participants’ background and frailty status

Item	Group				Bonferroni test
	Healthy	Pre-Frail	Frail		
	Number of people (%)				
	50 (31.8%)	76 (48.4%)	31 (19.7%)		
Background					
Sex (male)	27 (54.0)	41 (53.9)	11 (35.5)	0.856^b)^	
Age (years) (mean±SD)	72.7±4.1	75.1±6.2	79.8±6.2	<0.000^a^	d) e)
Drinking	13 (26.0)	9 (11.8)	6 (19.4)	0.123^b)^	H<P<F
Smoking	4 (8.0)	5 (6.6)	2 (6.5)	0.946^b)^	
Driving	32 (68.1)	46 (66.7)	17 (58.6)	0.674^b)^	
Economic difficulty in life	11 (22.9)	32 (42.1)	20 (64.5)	0.008^b)^	d) P<F
MMSE (points)	28.1±2.3	27.0±2.9	25.0±3.9	<0.000^a^	d) e) H>P>F
MNA (points)	26.8±2.1	26.9±11.6	23.6±4.0	<0.000^a^	e) H>F
PH Diabetes pathophysiology, treatment methods, complications					
Duration of diabetes (years)	12.5±6.3	14.4±7.8	15.4±7.7	0.222^a)^	
Height (cm)	159.1±8.7	157.1±9.1	152.0±9.2	0.010^a)^	d) e) H>P>F
Weight (kg)	61.9±11.0	59.0±12.8	53.2±12.6	0.007^a)^	e) H>F
BMI	24.5±4.0	23.9±4.3	22.8±4.2	0.380^a)^	
HbA1c (%)	8.3±8.9	7.6±1.3	7.4±1.1	0.210^a)^	
Insulin use	8 (16.0)	24 (31.6)	11 (35.5)	0.084^b)^	
DPP-4 use	28 (56.0)	40 (52.6)	18 (58.1)	0.858^b)^	
GLP-1 use	15 (30.0)	22 (28.9)	6 (19.4)	0.530^b)^	
SU use	18 (36.0)	23 (30.3)	6 (19.4)	0.282^b)^	
Pio use	3 (6.0)	6 (7.9)	0 (0.0)	0.280^b)^	
BG use	10 (20.0)	15 (19.7)	3 (9.7)	0.416^b)^	
Neurosis	22 (44.0)	42 (55.3)	11 (68.8)	0.060^b)^	
Retinopathy	16 (32.0)	20 (26.3)	10 (32.3)	0.728^b)^	
Nephropathy	25 (50.0)	44 (57.0)	18 (58.1)	0.647^b)^	
Hypertension	29 (58.0)	48 (63.2)	22 (71.0)	0.501^b)^	
Dyslipidemia	42 (84.0)	61 (80.3)	18 (58.1)	0.017^b)^	d) P>F
Coronary artery disease	8 (16.0)	10 (13.2)	7 (22.6)	0.482^b)^	
Cerebrovascular disease	3 (6.0)	8 (10.5)	6 (19.4)	0.170^b)^	
Arteriosclerosis obliterans	3 (6.0)	9 (11.8)	7 (22.6)	0.084^b)^	
Vision status					
History of cataract OP	48 (96.0)	72 (94.7)	30 (96.8)	0.882^b)^	
Vision awareness	16 (32.0)	20 (26.3)	13 (41.9)	0.283^b)^	
Good vision (log value)	0.38±0.3	0.45±0.3	0.48±0.3	0.242^a)^	
Poor vision (log value)	0.43±0.2	0.52±0.2	0.52±0.2	0.068^a)^	
Presence or absence of simple vision impairment (cutoff: 0.5)	19 (38.0)	48 (63.2)	18 (58.1)	0.019^b)^	d) P>F
Use of glasses	26 (52.0)	38 (50.0)	14 (45.2)	0.834^b)^	
Hearing status					
Poor hearing value	36.4±14.2	42.5±13.9	46.1±15.4	0.001^a)^	c) e) H<P, H<F
Finger rub test hearing loss	13 (26.5)	37 (49.3)	14 (46.7)	0.034^b)^	d) P>F
WHO hearing loss	9 (18.4)	32 (42.1)	11 (35.5)	0.022^b)^	d) P>F
Subjective awareness of hearing loss	11 (22.0)	21 (27.6)	8 (25.8)	0.838^b)^	
Indication of hearing loss	2 (4.0)	20 (26.3)	9 (29.0)	0.003^b)^	d) P<F
Use of hearing aids	2 (4.0)	2 (2.6)	3 (9.7)	0.272^b)^	

(Mean±SD or n [%]) a) Kruskal-Wallis test, b) χ^2^ test. Simple regression analysis using the total OSHPE score as the dependent variable Bonferroni test: c) Healthy-Prefrail, d) Prefrail-Frail, e) Healthy-Frail Healthy: H, Prefrail: P, Frail: F **MNA** (Mini Nutritional Assessment), **MMSE** (Mini-Mental State Examination), **BMI** (body mass index), **HbA1c** (glycated hemoglobin), **DPP** (dipeptidyl peptidase-4), **GLP** (glucagon-like peptide 1), **SU **(sulfonylurea), **Pio** (pioglitazone), **BG** (biguanide), **OP** (surgery), **WHO** (World Health Organization), **SD** (standard deviation)

**Table2:** Multiple regression analysis with the total OSHPE score as the dependent variable

	Model 1					Model 2					Model 3			
	B	Standard error	β	*p*-value		B	Standard error	β	*p*-value		B	Standard error	β	*p*-value
Neurosis											0.284	0.178	0.117	0.114
Dyslipidemia											–0.494	0.210	–0.171	0.020
Cerebrovascular disease											0.554	0.291	0.140	0.058
MMSE											–0.054	0.030	–0.140	0.074
MNA											–0.169	0.057	–0.214	0.004
Economic difficulty in life											0.349	0.149	0.172	0.020
Poor hearing ear value	0.020	0.006	0.266	0.0010							0.015	0.006	0.176	0.018
Simple vision cutoff 0.5						0.226	0.194	0.093	0.245		–0.034	0.181	–.0014	0.850

Model 1 Simple regression Model 2 Simple regression Model 3 Included and adjusted for age and sex Independent variables: Poor hearing ear value, simple vision cutoff 0.5, neurosis, dyslipidemia, cerebrovascular disease, MMSE score, MNA score, and economic difficulty in life.

## Data and/or Code Availability

The datasets generated during and/or analyzed during the current study are available from the corresponding author on reasonable request.
